# Neurological manifestations in chronic hepatitis C patients receiving care in a reference hospital in sub-Saharan Africa: A cross-sectional study

**DOI:** 10.1371/journal.pone.0192406

**Published:** 2018-03-07

**Authors:** N. Y. Mapoure, M. N. Budzi, S. A. F. B. Eloumou, A. Malongue, C. Okalla, H. N. Luma

**Affiliations:** 1 Department of Clinical Sciences, Faculty of Medicine and Pharmaceutical Sciences, University of Douala, Douala, Cameroon; 2 Department of Internal Medicine, Douala General Hospital, Douala, Cameroon; 3 Faculty of Health Sciences, University of Buea, Buea, Cameroon; 4 Department of Biological Sciences, Faculty of Medicine and Pharmaceutical Sciences, University of Douala, Douala, Cameroon; 5 Department of Internal Medicine, Faculty of Medicine and Biomedical Sciences, University of Yaoundé I, Yaounde, Cameroon; Centers for Disease Control and Prevention, UNITED STATES

## Abstract

**Background:**

Chronic hepatitis C infection is a major public health concern, with a high burden in Sub-Saharan Africa. There is growing evidence that chronic hepatitis C virus (HCV) infection causes neurological complications. This study aimed at assessing the prevalence and factors associated with neurological manifestations in chronic hepatitis C patients.

**Methods:**

Through a cross-sectional design, a semi-structured questionnaire was used to collect data from consecutive chronic HCV infected patients attending the outpatient gastroenterology unit of the Douala General Hospital (DGH). Data collection was by interview, patient record review (including HCV RNA quantification, HCV genotyping and the assessment of liver fibrosis and necroinflammatory activity), clinical examination complemented by 3 tools; Neuropathic pain diagnostic questionnaire, Brief peripheral neuropathy screen and mini mental state examination score. Data were analysed using Statistical package for social sciences version 20 for windows.

**Results:**

Of the 121 chronic hepatitis C patients (51.2% males) recruited, 54.5% (95% Confidence interval: 46.3%, 62.8%) had at least one neurological manifestation, with peripheral nervous system manifestations being more common (50.4%). Age ≥ 55 years (Adjusted Odds Ratio: 4.82, 95%CI: 1.02–18.81, p = 0.02), longer duration of illness (AOR: 1.012, 95%CI: 1.00–1.02, p = 0.01) and high viral load (AOR: 3.40, 95% CI: 1.20–9.64, p = 0.02) were significantly associated with neurological manifestations. Peripheral neuropathy was the most common neurological manifestation (49.6%), presenting mainly as sensory neuropathy (47.9%). Age ≥ 55 years (AOR: 6.25, 95%CI: 1.33–29.08, p = 0.02) and longer duration of illness (AOR: 1.01, 1.00–1.02, p = 0.01) were significantly associated with peripheral neuropathy.

**Conclusion:**

Over half of the patients with chronic hepatitis C attending the DGH have a neurological manifestation, mainly presenting as sensory peripheral neuropathy. Routine screening of chronic hepatitis C patients for peripheral neuropathy is therefore necessary, with prime focus on those with older age and longer duration of illness.

## Introduction

Hepatitis C is a major public health problem affecting greater than 185 million people worldwide [1]. It is estimated that 3–4 million people are infected annually, and 350,000 people die yearly from hepatitis C related complications [2]. Several studies have shown that 90% of chronically infected HCV infected patients live in low and middle income countries [1,3,4]. Africa has the estimated regional seroprevalence (5.3%), with Sub-Saharan Africa being greatly affected [5,6]. In Cameroon varying reports of hepatitis C seroprevalence exist, with country estimates of up to 13%, second only to Egypt with the highest estimated seroprevalence (17%) in the continent and in the world [5].

Amongst those infected with HCV, 55–80% become chronically infected, but most may remain asymptomatic until complications set in [7]. Prior to onset of signs and symptoms of advanced liver disease, several extrahepatic manifestations (EHMs) have been reported in up to 74% of infected patients [8]. EHMs may represent the first indication of chronic HCV infection and are capable of aggravating the clinical spectrum of hepatic disease or even dominating the clinical scenario [2]. Amongst the EHMs, neurological manifestations are estimated to occurring in up to 50% of HCV infected patients [9–11].

The most studied HCV associated neurological conditions include: peripheral neuropathy, cognitive impairment and cerebrovascular accidents [10,12,13]. Peripheral neuropathy is the commonest and best established HCV related neurological complication, affecting more than half of infected patients in some populations [12,13]. The pathogenesis of these manifestations remain largely speculative. Implicated mechanisms include direct neurotoxicity, metabolic and neurotransmitter imbalance, chronic inflammation and immune mediated responses [14]. Neurologists are increasingly faced with diagnosing or even predicting various neurological complications of HCV infection and/or its treatment. These complications may interfere with effective disease management, contribute to increased morbidity, disability and decreased quality of life [15].

A few studies have been carried out in developed countries on HCV related neurological manifestations. Egypt is the only African country with published information on these manifestations. Manal et al. reported the prevalence of peripheral neuropathy as 67.5% in a group of Egyptian HCV infected treatment naïve patients [13]. The most reported factors associated with neurological manifestations in HCV infection are cryoglobulinaemia, advanced age and duration of illness [16–18]. Despite the high burden of Hepatitis C in sub-Saharan Africa, there is a dearth of data on the effect of chronic HCV infection on the nervous system in this region. An updated knowledge on the burden of these manifestations in HCV infected patients will serve as a standpoint for assessing the need for routine neurological assessment of chronic HCV infected patients. It will also highlight the need for multidisciplinary management of these patients. Thus the present study was designed to determine the prevalence and factors associated with neurological manifestations in chronic hepatitis C infected patients.

## Materials and methods

### Study design, setting and participants

This cross-sectional study was carried out in the outpatient gastroenterology unit of the Douala General Hospital (DGH) from January to March 2017. DGH is a tertiary health facility in Douala, the economic capital of Cameroon. This hospital hosts many services and units amongst which are a gastroenterology and a neurology unit. The gastroenterology unit of DGH is one of the national treatment centres for Hepatitis B and Hepatitis C infected patients. DGH also has a well-equipped laboratory where most baseline investigations for Hepatitis C infection diagnosis and management are done. Following anti-HCV positive testing, further evaluation is proposed including HCV RNA quantification, HCV genotyping and the assessment of liver fibrosis with the Fibrotest (BioPredictive, Paris, France) and necroinflammatory activity with Actitest (BioPredictive). Cryoglobulin testing is not routinely done in the hospital laboratory.

The participants during the study period benefited from a concurrent research where their serum cryoglobulin was analysed, and the results placed in their records. The steps used in detection of cryoglobulins follows the practical protocol proposed by the National College of Hospital Biochemistry “cryoglobulin” working group [19]. Cryoglobulinaemia was classified according to the criteria described by Brouet et al [20].

All consenting patients followed up for chronic HCV infection (treatment naïve, receiving or completed treatment) at the gastroenterology outpatient unit were included. Patients aged less than 18 years, or those with hepatocellular carcinoma were excluded.

### Study procedure and data collection

All eligible participants underwent an interview, a general physical examination (including weight and height measurement) and a complete neurological examination. Participant’s files were reviewed for Hepatitis C history, treatment status, complications and results of investigations. A semi-structured questionnaire was used to record the following; socio-demographic data(age, gender, residence, occupation, insurance status and level of education), co-morbidities, hepatitis C history and assessment (date and circumstance of diagnosis, potential risk factors, complications, HCV genotype, viral load, METAVIR fibrosis and activity scores), neurologic symptom review, physical examination findings, neurological examination findings and results of investigations.

### Neurological assessment

Neurological assessment was done by a detailed neurological examination under different domains; higher mental function (cognitive assessment mainly), cranial nerve assessment, motor function assessment (muscle bulk, tone and power), reflexes (deep tendon and superficial reflexes), sensory testing (pain, light touch proprioception and vibratory perception), coordination and gait. The mini mental state examination (MMSE) tool was used for the diagnosis of cognitive impairment, while the Neuropathic Pain Diagnostic Questionnaire (NPDQ) and Brief Peripheral Neuropathy screen (BPNS) were used for diagnosis of neuropathic pain and peripheral neuropathy respectively. The NPDQ (also known as the Douleur Neuropathique 4 questionnaire) is a validated tool for diagnosis of neuropathic pain, consisting of ten items (7 interview based and 3 based on clinical examination). It has a sensitivity of 83% and a specificity of 90% [21]. The BPNS tool is a valid screening tool used in the context of HIV infection, and is simple enough to be applicable in resource limited settings [22].

### Operational definitions

Chronic Hepatitis C: The presence of detectable HCV RNA, currently or in the past (i.e. receiving treatment or completed treatment).

Duration of illness: This was defined as the period from diagnosis of HCV infection to the date of interview for patients naïve to treatment and those receiving treatment. For those who had completed treatment, it was the period between diagnosis and date of sustained virological response confirmation.

Paraclinical Investigations: This included haematological, biochemical, virological and histological investigations in patient’s record. The results of the most recent investigations done in the last 6 months (for haematological and biochemical investigations) and 1 year (for viral load and histological investigations) were used.

Viral load was classified as high (≥ 600,000IU/l) or low (< 600,000IU/l).

Significant fibrosis: METAVIR fibrosis score of ≥F2.

Significant activity: METAVIR activity score of ≥A2.

Alcohol intake: This was assessed by a Yes/No answer to the question ‘do you drink alcohol?’

Couple; cohabiting or married. Alone; single, divorced or widowed.

Neurological Manifestations: Syndromes of neurologic dysfunction and specific neurological diseases.

Cognitive impairment: MMSE score less than or equal to 24

25–30: No cognitive impairment.
20–24: Mild cognitive impairment.
15–19: Moderate cognitive impairment
<15: Severe cognitive impairment.

Cognitive domain impairment: A score of less than half of the total score in that domain of the MMSE.

Neuropathic Pain: A NPDQ score of greater than 4 will be used for diagnosis of neuropathic pain.

Peripheral Neuropathy: One bilateral objective finding (loss of vibration perception and abnormal ankle deep tendon reflexes), using the BPNS tool.

Symptomatic peripheral neuropathy: The presence of at least one bilateral objective finding and a subjective sensory neuropathy grade greater than 0 using the BPNS tool.

### Statistical analysis

Data analysis was done using Statistical Package for Social Sciences (SPSS) version 20 software. Frequencies and percentages were computed for categorical variables and mean (or median) and standard deviation (or interquartile range) for continuous variables. Associations between exposure variables and presence or not of neurological manifestations were assessed using chi’s square or Fisher’s exact tests and binary logistic regression where appropriate. Exposure variables with p-values<0.10 were further tested through multivariate logistic regression to account for possible confounding. A p value<0.05 was considered statistically significant.

### Ethical considerations

Ethical approval to conduct the study was obtained from the institutional review board of the Faculty of health sciences, University of Buea, Cameroon. Participants had study carefully explained to them and participation was voluntary. All participants provided written consent. Apart from the inconvenience of the time spent in answering questions and being examined, participants were not exposed to any undue risk. Neurological diagnosis was explained to participants and they were linked to the neurology unit for further care when necessary.

## Results

### General Characteristics of participants

#### Sociodemographic characteristics and medical history

**Fig 1:**
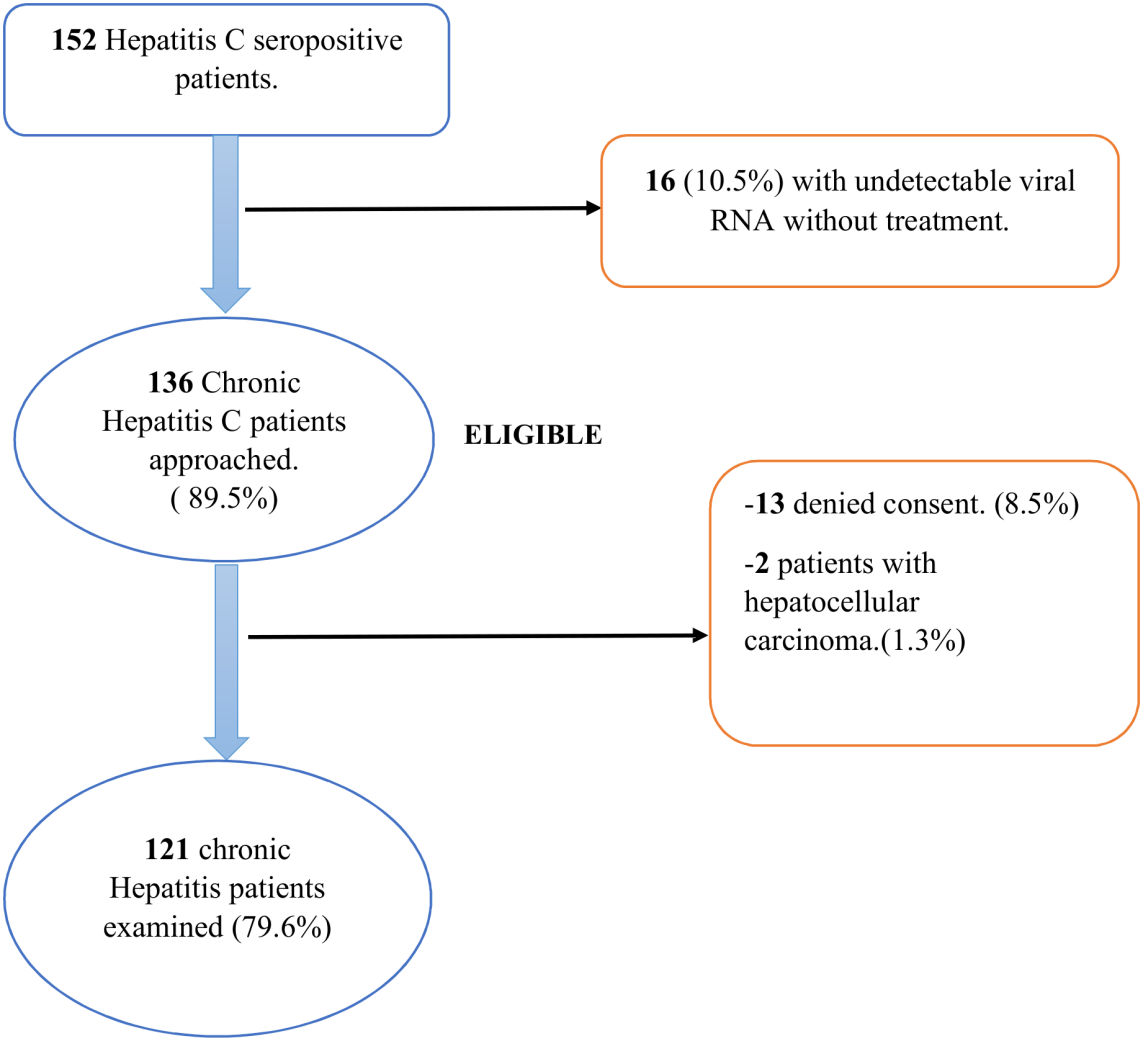
Flow chart of patient enrollment.

Of 136 chronic hepatitis C patients seen during the study period, 121 were included in the study, as shown in Fig 1. The age of participants ranged from 33 to 74 years with a mean age of 59.1±9.0 years. Males were more represented (51.2%), without any significant difference. At least one comorbidity was present in 53.7% (n = 65) of the population. Diabetes was present in 17.4% (n = 21) of participants. Among the patients studied, 2.5% (n = 3) each were co-infected with either HIV or Hepatitis B virus. The median duration of HCV infection was 32 months (IQR 12–71). Majority of the participants were asymptomatic at diagnosis, 63.6% (n = 83). At the time of the study, 55.4% (n = 67) of the population were treatment naïve. Sociodemographic characteristics and medical history of study participants are shown in Tables 1 and 2 repectively.

**Table 1:** Sociodemographic characteristics of study participants.

Variable	Frequency	Percentage (%)
**Age (years)**		
<35	3	2.5
35–44	7	5.8
45–54	17	14.0
55–64	64	52.9
≥65	30	24.8
mean ± SD	59.1 ± 9.0	
**Sex**		
Male	62	51.2
Female	59	48.8
**Marital status**		
Couple	90	74.4
Alone	31	25.6
**Profession**		
Unemployed	72	59.5
Employed	49	40.5
**Level of Education**		
No formal education	3	2.5
Primary	19	15.7
Secondary	54	44.6
Tertiary	45	37.2
**Insurance coverage**		
No	101	83.5
Yes	20	16.5

SD: Standard deviation.

**Table 2:** Medical history of study participants.

Variable	Frequency	Percentage (%)
**Comorbidities(n = 121)**		
Hypertension	38	31.4
Diabetes	21	17.3
History of TB.	8	6.6
CKD	5	4.1
HIV co-infection	3	2.5
Hepatitis B co-infection	3	2.5
Traditional medication use	81	66.9
Alcohol use	33	27.3
Cigarette smoking	7	5.8
**Hepatitis C History**		
**Duration of illness, months (N = 121)**		
<60	80	66.1
≥60	41	33.9
Duration of illness(median, IQR)	32 (12–71)	
**Circumstance of diagnosis(N = 121)**		
Assymptomatic	83	63.6
Symptomatic	28	31.4
**Treatment status (N = 121)**		
Never treated	67	55.4
Receiving treatment	14	11.6
Treatment failed/interrupted	5	4.1
Treated with SVR.	35	28.9
**Treatment regimen (N = 54)**		
IFN based	15	27.8
IFN free	39	71.4
**Complications (N = 121)**		
Cirrhosis	21	17.3

TB: Tuberculosis; CKD: Chronic kidney disease; IQR: Interquartile range; SVR: sustained virological response; IFN: interferon.

#### Clinical and paraclinical characteristics

The mean BMI of the participants was 27.5 ±4.2 kg/m^2^. A great majority of the population were either obese or overweight, 72.7% (n = 88). The commonest feature on general examination was conjunctival pallor in 6.6% (n = 8) of population. Anaemia and leucopenia were present in 36.6% (37/101) and 39% (39/100) of study participants respectively. Cryoglobulinaemia was present in 72.9% (70/96) of participants. Mixed cryoglobulinaemia made up the majority (92.8%) of cryoglobulin positive patients. The median viral load was 1405005 UI/ml (IQR 399934–3256655), with 66.7% (66/99) of patients having a high viral load. The most common genotype was genotype 1 present in 51.6% (47/91) of patients. Of the 87 participants with available fibrosis scores, 69.2% (n = 54) had significant fibrosis (Table 3).

**Table 3:** Laboratory characteristics of study participants.

Variable	Frequency	Percentage (%)
**Haematological parameters**		
**Haemoglobin, g/dl (N = 100)**		
<12 g/dl	37	36.6
**WBC count, per mm3 (N = 101)**		
<4000/mm3	39	39
**Platelet count, per mm3 (N = 101)**		
<100,000/mm3	8	7.9
**INR (N = 60)**		
>1	33	55
**Biochemical parameters.**		
**ALT, IU/l (N = 100)**		
≥40 IU/l	33	33
**AST, IU/l (N = 99)**		
≥40 IU/l	33	33.3
**Serum albumin, g/l (N = 32)**		
<35g/l	8	32.0
**Fasting blood glucose, g/l (N = 67)**		
≥1g/l	24	35.8
**Total cholesterol, g/l (N = 41)**		
≥2g/l	24	35.8
**LDL cholesterol, g/l (N = 35)**		
≥1g/l	14	34.1
**HDL cholesterol, g/l (N = 37)**		
<0.50g/l	16	51.4
**Creatinine, mg/l (N = 82)**		
≥13.0 mg/l	12	14.6
**Cryoglobulin (N = 96)**		
Negative	26	27.1
Positive	70	72.9
Type 1	5	7.1
Type 2	47	67.1
Type 3	18	25.7
**Virological parameters**		
**Viral Load, IU/ml (N = 99)**		
Low viral load (<600,000)	33	33.3
High viral load (≥ 600,000)	66	66.7
Viral load value (median, IQR)	1,405,005 (399,934–3,256,655)	
**Genotype (N = 91)**		
Genotype 1	47	51.6
Genotype 2	22	24.2
Genotype 4	22	24.2
**Histological parameters.**		
**Fibrosis METAVIR score (N = 87)**		
F0	10	12.8
F1	14	18.0
F2	21	26.9
F3	13	16.7
F4	20	25.6
**Activity METAVIR score (N = 52)**		
A0	12	23.1
A1	14	26.9
A1	17	32.7
A3	9	17.3

ALT: Alanine aminotransferase; AST: Aspartate aminotransferase; HDL: High density lipoproteins; INR: International normalized ratio; LDL: Low density lipoprotein; WBC: White blood cell.

### Prevalence and clinical patterns of neurological manifestations

The prevalence of neurological manifestations in the study population was 54.5% (95CI: 46.3–62.8%), with predominance of disorders in the peripheral nervous system, 50.4% (61/121). Peripheral neuropathy was quite common and was present in about half of the patients, 49.6% (60/121). Cognitive impairment affected less than a tenth of the population, 8.3% (10/121) (Table 4).

**Table 4:** Prevalence and clinical patterns of neurological manifestations.

Variable	Frequency	Percentage (%)
**Neurological manifestations**		
Yes	66	54.5
No	55	45.5
**Type of disorder**		
CNS disorder	13	10.7
PNS disorder	61	50.4
**CNS disorder**		
Cognitive impairment	10	8.3
Stroke	3	2.5
Oral myoclonus	1	0.8
**PNS disorder**		
Peripheral neuropathy	60	49.6
Cranial neuropathy	5	4.1
Carpal tunnel syndrome	1	0.8

CNS: Central nervous system; PNS: peripheral nervous system

#### Neurologic symptoms

**Fig 2:**
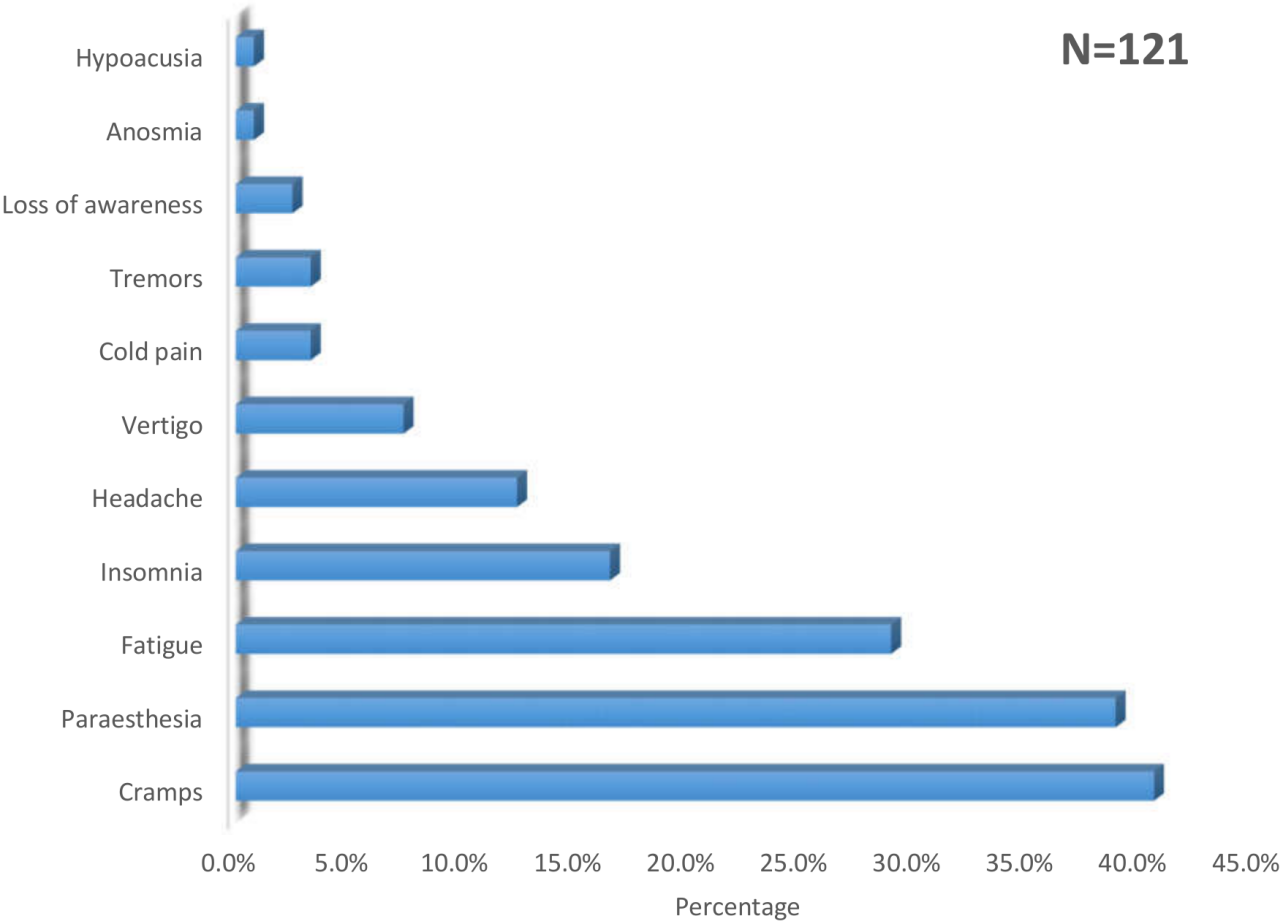
Neurological symptoms reported by chronic hepatitis C patients.

The most common symptom reported during the study period were cramps; 40.5% (n = 49), followed by paraesthesia in 38.8% (n = 47) and fatigue in 28.9% (n = 24). Paraesthesia was mainly reported as tingling in 23.1% (n = 28) and burning sensation in 22.3% (n = 27). This is summarised in Fig 2.

#### Neurological signs

The commonest neurological sign present on examination was abnormal vibration perception in 46.2% (n = 23), followed by a hypoactive ankle jerk reflex in 18.2% (n = 22). A cranial nerve abnormality was present in 4.1% (n = 5) of the population with unilateral facial palsy being the most frequent, 3.3% (n = 4). Neuropathic pain was present in 9.1% (n = 11) of the population.

#### Cognition assessment

The mean MMSE score was 27.0 ±2.8. All patients with cognitive impairment had mild cognitive impairment. On assessment of cognitive domains of all patients, there was no impairment in orientation or registration. The main cognition domain impaired was recall, 26.4% (32/121) followed by attention, 19% (26/121). Table 5 summarises the cognitive assessment findings.

**Table 5:** Cognitive assessment findings.

Variable	Frequency	Percentage (%)
**Overall cognition assessment**		
No cognitive impairment	110	91.7
Mild cognitive impairment	10	8.3
MMSE score (mean ± SD)	27.0 ± 2.8	
**Cognitive domain abnormality**		
Recall domain	32	26.4
Attention domain	26	19.0
Visual Construction domain	9	7.4
Language domain	1	0.8

#### Peripheral neuropathy assessment

Based on the brief peripheral neuropathy screen (BPNS), 58.3% (35/60) of participants with peripheral neuropathy were symptomatic, with 60% (21/35) having mild symptoms. The main form of peripheral neuropathy was sensory neuropathy (n = 58, 96.6%). On vibration testing 10.7% (n = 13) had absent vibration sensation bilaterally (Table 6).

**Table 6:** Peripheral neuropathy assessment findings.

Variable	Frequency	Percentage (%)
**Peripheral neuropathy subclass**		
Symptomatic	35	28.9
Sensory	58	47.9
Sensori-motor	1	0.8
Motor	1	0.8
Upper limb	2	1.7
Lower limb	58	47.9
**Subjective Sensory grade**		
0 (No sensory neuropathy)	86	71.1
1 (Mild sensory neuropathy)	21	17.3
2 (Moderate sensory neuropathy)	10	8.3
3 (Severe sensory neuropathy)	4	3.3
**Vibration perception score**		
0 (felt for >10 seconds)	65	53.7
1 (felt for 6–10 seconds)	29	24.0
2 (felt for ≤ 5 seconds)	14	11.6
3 (not felt)	13	10.7
Perception time, seconds (mean ±SD)	7.9±3.6	
**Ankle reflex grade**		
0 (Absent)	2	1.7
1 (Hypoactive)	22	18.2
2 (Normal)	97	80.2

### Factors associated with neurological manifestations

In univariate analysis, the following factors with neurological manifestations in chronic hepatitis C patients; age ≥55 years (p = 0.002), being overweight (p = 0.007), longer duration of infection (p = 0.016), fasting blood glucose ≥1g/l (p = 0.04), total cholesterol ≥2g/l (p = 0.04) and serum albumin < 35g/l (p = 0.02) and, high viral load was associated with neurological manifestations (p = 0.05). (Tables 7 and 8).

**Table 7:** Clinical factors associated with neurological manifestations in hepatitis C infected patients.

	Neurological manifestation n(%)		Odds Ratio	95% CI	P value
	No	Yes			
**Age (years)**					
<55	20 (74.1)	7 (25.9)	REF		
≥55	35 (37.2)	59 (62.8)	4.82	1.85–12.54	**0.002**
**Gender**					
Male	29 (46.8)	33 (53.2)	REF		
Female	26 (44.1)	33 (55.9)	1.11	0.54–2.28	0.76
**Marital Status**					
Alone	11 (35.5)	20 (64.5)	REF		
In Couple	44 (48.9)	46 (51.1)	0.57	0.25–1.13	0.20
**Years of Education**					
< 7 years	6 (27.3)	16 (72.7)	0.37	0.13–1.04	0.06
≥ 7 years	49 (50)	49 (50)	REF		
**BMI**					
Normal	19 (61.3)	12 (38.7)	REF		
Underweight	1 (100)	0 (0.0)	0.00		1.00
Overweight	17 (30.9)	38 (69.1)	3.54	1.41–8.89	**0.007**
Obese	16 (50.0)	16 (50.0)	1.58	0.58–4.31	0.37
**Duration of illness**					
Duration of infection (months)			1.01	1.00–1.02	**0.016**
**Treatment status**					
-Never treated	30 (44.8)	37 (55.2)	REF		
-Receiving treatment	7 (50)	7 (50)	0.81	0.26–2.57	0.72
-Failed/interrupted treatment	3 (60.0)	2 (40.0)	0.54	0.85–3.45	0.51
-Completed treatment with SVR	15 (42.9)	20 (57.1)	1.08	0.47–2.47	0.83
**Treatment regimen**					
Interferon free	18 (46.2)	21 (53.8)	REF		
Interferon based	7 (46.7)	8 (53.3)	0.98	0.30–3.23	0.97
**Complications**					
Cirrhosis	8 (38.1)	13 (61.9)	1.47	0.56–3.85	0.43

**Table 8:** Paraclinical factors associated with neurological manifestations in hepatitis C infected patients.

	Neurological manifestation n(%)		Odds Ratio	95% CI	P value
	No	Yes			
Haemoglobin (g/dl)					
**<12**	19 (51.4)	18 (48.6)	1.88	0.183–4.28	0.13
WBC count (cells/mm^3^)					
**<4000**	16 (41.0)	23 (59.0)	0.75	0.45–1.25	0.27
INR					
**≥1**	10 (30.3)	23 (69.7)	1.58	0.54–4.60	0.40
ALT (IU/l)					
**≥40**	11 (33.3)	22 (66.7)	1.94	0.81–4.62	0.13
AST (IU/l)					
**≥40**	12 (36.4)	21 (63.6)	1.65	0.70–3.88	0.25
Fasting Blood glucose					
**≥1g/l**	6 (25)	18 (75)	3.14	1.05–9.45	**0.041**
Total Cholesterol (g/l)					
**≥2g/l**	2 (14.3)	12 (85.7)	5.57	1.04–29.79	**0.045**
LDL Cholesterol					
**≥1g/l**	7 (38.9)	11(61.1)	1.40	0.36–5.35	0.63
HDL Cholesterol (g/l)					
**<0.50 g/l**	7 (43.8)	9 (56.2)	1.26	0.34–4.74	0.73
Albumin (g/l)					
**< 35g/l**	5 (62.5)	3 (37.5)	8.33	1.39–49.87	**0.020**
Creatinine (mg/l)					
**≥ 13.0 mg/l**	5 (41.7)	7 (58.3)	0.93	0.27–3.24	0.91
Cryoglobulinaemia					
**Negative**	12 (46.2)	14 (53.8)	REF		
**Positive**	34 (48.6)	36 (51.4)	0.91	0.37–2.24	0.83
Viral load (IU/ml)					
**Low (< 600,000)**	19 (57.6)	14 (42.4)	REF		
**High (≥ 600,000)**	24 (36.4)	42 (63.6)	2.37	1.01–5.57	**0.047**
Genotype					
**Genotype 1**	21 (44.7)	26 (55.3)	1.13	0.49–2.58	0.77
**Genotype 2**	10 (45.5)	12 (54.5)	1.04	0.39–2.72	0.94
**Genotype 4**	11 (50.0)	11 (50.0)	0.82	0.31–2.13	0.68
Fibrosis grade					
**Insignificant (<F2)**	11 (45.8)	13 (54.2)	REF		
**Significant (≥F2)**	25 (46.3)	29 (53.7)	0.98	0.37–2.58	0.97
Activity grade					
**Insignificant (< A2)**	13 (50.0)	13 (50.0)	REF		
**Significant (≥ A2)**	11 (42.3)	15 (57.7)	1.36	0.46–4.07	0.58

In multivariate analysis, age ≥ 55 years (AOR: 4.82, 95%CI: 1.02–18.81, p = 0.02), longer duration of illness (AOR: 1.012, 95%CI: 1.00–1.02, p = 0.01) and high viral load (AOR: 3.40, 95% CI: 1.20–9.64, p = 0.02) were independently associated with neurological manifestations (Table 9).

**Table 9:** Predictors of neurological manifestations in chronic hepatitis C patients.

Variable category	AOR(95% CI)	p-value
**Age ≥55 years**	4.82 (1.02–18.81)	**0.023**
**Duration of illness (months)**	1.012 (1.00–1.02)	**0.014**
**Overweight**	3.09 (0.92–10.43)	0.07
**< 7 years of education**	2.23 (0.67–7.42)	0.19
**High viral load**	3.40 (1.20–9.64)	**0.022**
**FBS ≥ 1g/l**^a^	NA	NA
**Albumin <35g/l**^a^	NA	NA
**Total Cholesterol ≥2g/l**^a^	NA	NA

^a^ Not included in multivariate analysis because of too few values.

AOR: Adjusted odds ratio; FBS: fasting blood sugar; CI: Confidence interval; NA: Not applicable.

### Factors associated with peripheral neuropathy

In multivariate analysis, age ≥ 55 years (AOR: 6.25, 95%CI: 1.33–29.08, p = 0.02) and longer duration of illness (AOR: 1.01, 1.00–1.02, p = 0.01) were identified as factors independently associated with peripheral neuropathy (Table 10).

**Table 10:** Predictors of peripheral neuropathy in chronic hepatitis C patients.

Variable	Univariate analysis		Multivariate analysis (adjusted for presence of diabetes)	
	p-value	OR (95%CI)	p- value	AOR (95%CI)
**Age ≥55 years**	0.002	4.72 (1.74–12.78)	**0.02**	6.25 (1.33–29.08)
**Duration of illness (months)**	0.01	1.01 (1.00–1.02)	**0.015**	1.01 (1.00–1.02)
**Presence of comorbidity**	0.08	1.90 (0.92–3.91)	0.26	1.90 (0.61–6.20)
**Overweight**	0.041	2.56 (1.04–6.33)	0.22	2.40 (0.59–9.77)
**AST ≥40 IU/l**	0.06	2.29 (0.97–5.41)	0.35	2.05 (0.46–9.11)
**ALT ≥ 40 IU/l**	0.049	0.42 (0.18–1.00)	0.66	1.38 (0.33–5.77)
**High viral load**	0.09	2.09 (0.89–4.89)	0.13	2.37 (0.77–7.27)

OR: Odds ratio, AOR: Adjusted odds ratio; CI: confidence interval; AST: Aspartate aminotransferase; ALT; Alanine aminotransferase.

## Discussion

In this study, we found that one in two patients had a neurological manifestation following chronic infection with hepatitis C virus. We also showed that the most common neurological manifestations were peripheral neuropathy and cognitive impairment. The most common neurologic sign on examination was abnormal vibration sense, while the most common symptom was limb cramps. Factors associated with neurological manifestations were; age≥ 55 years, longer duration of illness and high viral load (≥ 600,000IU/l). Specifically, for peripheral neuropathy, age ≥55years and longer duration of illness were identified as associated factors.

To the best of our knowledge, this is among the few studies documenting the overall prevalence and types of neurological manifestations in chronic hepatitis C. Most studies looked only at a unique disorder or partition of the nervous system. The prevalence of neurological manifestations in our study was 54.5%. This indicates that more than half of patients with chronic HCV have an additional morbidity burden that is somewhat neglected. This prevalence found in our study is consistent with the estimate of 50% reported in three systematic reviews [9–11].

In this study, peripheral neuropathy was the most common neurological manifestation, with a prevalence of 49.6% using the brief peripheral neuropathy screening (BPNS) tool. Our prevalence is similar to the 45% clinical neuropathy reported by Manal *et al*. in Egypt [13], and 43.5% by Yoon *et al*. in Germany [23]. However, our report was divergent with other studies carried out in Egypt by Al kafrawy *et al*., Abul Hassan *et al*., Abo Al-Soud *et al*. and El Ghoneimy *et al*. reporting prevalence estimates between 15.6 and 30% [24–27]. Other studies in non-African countries recorded even lower prevalence estimates of 9%, 10.6% and 14% in France, Italy, and Brazil respectively [28–30]. This wide variability could be attributed to the difference in sample sizes and study designs. Most of these studies used small sample sizes of less than 100 patients. Furthermore, these studies employed strict exclusion criteria, excluding patients with renal failure, diabetes, HIV and Hepatitis B co-infection and patients on treatment, who were otherwise included in our study. Secondly, this can be explained by the difference in methods and tools used for peripheral neuropathy diagnosis. For example, Cacoub *et al*. [28] used only symptoms reported in patient’s records to denote neuropathy, which has a lower diagnostic accuracy. The BPNS used in our study assesses for both signs and symptoms of peripheral neuropathy. Our findings are lower than the 58% reported by Telez-Zenteno *et al*. in Mexico [31] and 68% by Biasiotta *et al*. in Italy [32]. This could be explained by the fact that neuropathy assessment was done by electrophysiology, the reference standard test, which has better diagnostic accuracy compared to clinical examination. Moreover, Biasiotta *et al*. [32] used a selected group of HCV patients; cryoglobulin positive patients, which can also account for the high prevalence.

The predominance of sensory neuropathy in our study (96.7% of peripheral neuropathy) ties with previous reports by Manal *et al*. (94.4% of peripheral neuropathy was sensory), Al Kafrawy *et al*. (100% sensory) in Egypt and Biasiotta *et al*. (96% sensory) [13,24,32]. This predominance of sensory peripheral neuropathy is in line with Sterling and Bralow, who demonstrated that sensory deficiencies are more common than motor loss, and that sensory symptoms may persist for months to years before any motor deficit becomes clinically evident [33].

In our study, the prevalence of cognitive impairment was 8.3%. Our prevalence is close to the 13% reported by Mc Andrews *et al*. in Canada [34] and 16% by Kramer *et al*. in Austria [35]. Nonetheless, our prevalence is lower than the 38% and 33% observed by Hilsabeck *et al*. and Fontana *et al*. respectively in USA [36,37]. A possible reason for this differences isthe different tools used for assessment of cognitive impairment. Most of the studies used neuropsychological batteries with several tests for different cognitive domains. The mini mental state examination used in our study has a lower sensitivity for cognitive impairment and assesses fewer domains compared to the detailed neuropsychological used in the other studies. Secondly, the contrast could be due to the heterogeneity in comorbidities of these patients. The proportion of cirrhotic patients in the study by Fontana *et al*. and Hilsabeck *et al*. were 38% and 51% respectively [36,37], compared to the 17% in our study.

The main cognitive domains impaired in chronic hepatitis C patients in our study were recall and attention using the MMSE. Our findings corroborate those of Ayman *et al*. in Egypt and Hilsabeck *et al*. [36,38]. Contrary to this, a study in Canada reported impaired learning efficiency and intact attention and verbal recall in Hepatitis C patients [34]. Forton *et al*. in London rather found that concentration and psychomotor speed as the main domains impaired [39]. This variation is most likely due to the use of different neuropsychological tests for the various domains in each study. This pattern of cognitive deficits exhibited by HCV patients is most consistent with frontal-subcortical dysfunction and is similar to that reported in patients with HIV [15].

The commonest neurological signs observed in our study were abnormal vibration sense and hypoactive Achille’s tendon reflex, concurring with Yoon *et al*.’s findings in Germany [23]. Neuropathic pain was present in 9.1% of our study population, assessed by the NPDQ tool. Our proportion is lower than the 43.7% reported in a study in Egypt and 42% reported in Italy [13,32]. This difference is most likely due to the difference in tools used for neuropathic pain assessment and the heterogeneity in populations studied. Unlike our study, Manal et al. used the Self-reported Leeds assessment neuropathy screen (SLANSS) [13]. Using the NPDQ tool, Biasiotta *et al*. in Italy had a higher proportion probably because they studied only cryoglobulin positive HCV patients [32].

The most common symptoms reported in our study were limb cramps (40%), paraesthesia (38.8%) and fatigue (28.9%). The proportion of patients with paraesthesia is congruent with the 38% and 34.6% reported in studies in Germany and Brazil [23,30]. However, contrary to our findings, cramps were reported in a lower proportion (11%) of the population. This could be due to the difference in age groups represented in these studies.

The predictors of neurological manifestations in our study were; age≥ 55years, longer duration of illness and a high viral load. Some authors have already noted that older age is a major risk factor for clinical and biological extrahepatic manifestations of chronic hepatitis C [40]. In keeping with these, we found an association between older age and neurological manifestations of HCV. Increasing age has been recognized as a risk factor for many neurological disorders, probably reflecting the limited regenerative capacity of the nervous system after any insult [41].

The significant relation between neurological manifestations and high viral load in our study supports the mechanism that Hepatitis C virus may have direct neurotoxic effects. The exact mechanism by which hepatitis C virus may enter the CNS is not known, however it is postulated that HCV may cross the blood brain barrier via infected monocytes using a ‘trojan horse mechanism’ [42].

Specifically, we observed that age above 55 years was associated with peripheral neuropathy. This is in accordance with previous reports in Egypt, Mexico and Italy [13,26,31,32], who reported older age as a predictor of peripheral neuropathy. Patients aged greater than 55 years were 6 times more likely to develop peripheral neuropathy than their younger counterparts in our study.

Longer duration of illness was also found as a predictor of peripheral neuropathy in our study. This is contrary to reports by Santoro et al and Yoon et al [23,29]. Nevertheless, our report is analogous to that of Manal *et al*. in Egypt, and Biasiotta *et al*. in Italy. It is not immediately clear to us why this difference exists [13,32].

In multivariate analysis, there was no significant association between viral load level and peripheral neuropathy. This contrasts the report of Manal *et al*. who observed a significant relationship between peripheral neuropathy and high viral load [13]. However our report concurs findings of studies carried out in Italy and Germany [23,29]. This difference could be due to the wide variation of viral genotypes represented in each geographical region. Moreover, our findings support the hypothesis that HCV related peripheral neuropathy results from virus triggered immune mediated mechanisms rather than from direct nerve infection and insitu replication [43]. In contrast to the brain, there is currently no evidence for peripheral nerves as permissive sites for HCV replication [10].

No association was found between cryoglobulinaemia and peripheral neuropathy in our study, despite the high prevalence of cryoglobulinaemia (72%), contrary to the report of two studies carried out in Egypt [13,26]. However, the pilot study carried out by Cacoub *et al*. in France [28]and that of Biasiotta *et al*. and Santoro *et al*. in Italy agree with our findings [29,32]. This lack of association is in keeping with the hypothesis that HCV related vascular nerve damage could be due to virus triggered immune mechanisms rather that long standing cryoglobulin precipitation.

A number of limitations have to be considered while interpreting these findings. This study was a hospital study carried out in a single treatment centre of a tertiary hospital, constraining the generalizability of our findings to all chronic hepatitis C patients who either do not seek care, or do not use tertiary health facilities. Furthermore, the mini mental state examination used for assessment of cognitive impairment lacks sensitivity compared to neuropsychological tests in the early diagnosis of mild cognitive impairment. Hence, this can lead to an underestimation of the actual burden of cognitive impairment in our study. However the MMSE remains a very useful tool easily used in clinical practice and is universally recognized to provide rapid information on the mental state of patients. Peripheral neuropathy was not assessed by neurophysiological methods which is the reference standard method for peripheral neuropathy diagnosis, thus missing cases of subclinical neuropathy. In addition, the heterogeneity of our study population including several potential confounders (HIV, diabetes, alcohol users, successfully treated and cryoglobulin positive patients) could affect the proportions and associations reported in our study. However the proportion of HIV and Hepatitis B infection were not significant, and presence of diabetes was adjusted in the multivariate models created, limiting its confounding effect. Other limitations include; the absence of an appropriate control group, and no correction for multiple comparison.

## Conclusion

Neurological manifestations in chronic hepatitis C infection, though underreported in sub-Saharan Africa, affects 1 out of 2 chronic hepatitis C patients in the Douala general hospital (54.5%), mainly presenting as sensory peripheral neuropathy. Older age, longer duration of illness and high viral load were independently associated with neurological manifestations in Chronic HCV infection. Factors found to be independently associated with peripheral neuropathy older age and longer duration of illness. Cognitive impairment was mild and affected mainly recall and attention. Routine screening of chronic hepatitis C patients for peripheral neuropathy is therefore necessary, with prime focus on those with older age and longer duration of illness. We also recommend that all patients presenting with a peripheral neuropathy, especially sensory peripheral neuropathy should be systematically screened for hepatitis C infection.

## Supporting information

S1 Dataset Minimal data set. (SAV)

S1 File Standard operating procedure for neurologic examination. (DOCX)
