# Supplementary material for: Neurological manifestations in chronic hepatitis C patients receiving care in a reference hospital in sub-Saharan Africa: A cross-sectional study
Source: PLoS One. 2018 Mar 7;13(3):e0192406. doi: 10.1371/journal.pone.0192406 (PMC5841655; doi:10.1371/journal.pone.0192406)
Supplement: S1 File — (DOCX) [file pone.0192406.s002.docx]

1. **NEUROLOGICAL EXAMINATION**

**Aim:** To screen for neurological disorders.

**Materials:**  Pen torch, tooth picks for pain sensation testing, reflex tendon hammer, 128MHz tuning fork and cotton wool.

**Procedure:**

***1. Cognitive assessment*:** This is assessed as steps of the mini mental state examination.

***2. Cranial nerve assessment:***

| *Cranial nerve I*  Check if nares are patent with pentorch.  With eyes closed, ask patient to sniff a mild stimulus; coffee, perfumed hand sanitizer, and identify odorant.  Test one nostril at a time. | *Cranial nerve II*  Each eye tested separately. If patient use glasses, they should put them on.  Patient is asked to read printed material at 30cm.  Visual fields: Face patient at a distance of 0.6 to 1m. Examiner closes right eye, while patient closes left. Keep eyes open and look directly at each other. Move your left arm out and away, keeping it equidistant from both of you. Wiggle finger and bring towards your nostril. You should be able to detect it at the same time. Begin with two inferior, then superior quadrants. Repeat for other eye. |
| --- | --- |
| *Cranial nerves II, IV, VI*  -Stand in front of patient. Ask patient to follow your finger while they keep their heads in position. Using your finger, trace an imaginary H in front of them, making sure your finger moves far enough out and up that you are able to see all eye movements.  Pupils: It helps to make room light dimmer. Using pentorch, shine light into one eye. Look for constriction of that eye (direct response). Remove light and re-expose it to same eye, though this time observing the other pupil (consensual response). Repeat for other eye. | *Cranial nerve V*  Sensory: Ask patient to close their eyes. Using a sharp implement (tooth pick), touch the right and left side of forehead, cheek area and jaw. Patient should indicate when he/she feels the prick.  With patient looking straight ahead, brush the wisp on the lateral aspect of the sclera. This should cause patient to blink (corneal reflex).  Motor: Palpate temporalis and masseter muscle as patient clenches and grinds teeth. |
| *Cranial nerve VII*  Look at patient’s face for any asymmetry (wrinkles of forehead, nasolabial folds and corners of mouth).  Ask patient to close eyes tightly (you should not be able to open patient’s eyelids), smile (corners of mouth should be the same height) and puff cheeks (both sides should puff equally and air should not leak from mouth). | *Cranial nerve VIII*  Stand behind patient and ask them to close eyes. Place fingers approximately 5 cm from one ear and rub them together. Patient should be able to hear. Repeat for other ear. |
|  | *Cranial nerve IX/X*  -Ask patient to open their mouth and say ‘ahhhh’ causing the soft palate to rise upward. Look at uvula. It should rise up straight in the midline. Use a tongue depressor if tongue obscures view.  Gag reflex: With mouth widely open, use tongue depressor to touch posterior pharynx. This should generate a gag in most patients. |
| *Cranial nerve XI*  -Place your hands on top of either shoulder and ask patient to shrug while you provide resistance.  Place your open left hand against the patient’s cheek and ask them to turn into your hand, while you provide resistance. Repeat on the other side. | *Cranial nerve XII*  -Ask patient to stick tongue straight out of their mouth. Tongue should me midline.  -Observe for atrophy or fasciculations. |

***3. Motor function assessment***

Muscle bulk and appearance: Using your eyes and hands, carefully examine the major muscle groups of upper and lower extremities. Muscle groups should appear symmetrically developed when compared to counterparts on the other side of the body. Look for tremors and fasciculation.

Muscle tone: Ask patient to relax joint to be tested. Carefully move the limb through the normal range of motion being careful not to maneuver it in any way that is uncomfortable or generates pain. Normal muscle generates some resistance to movement when a limb is moved passively by an examiner.

Muscle power: Gross limb power, tested for all four limbs, then tested for major muscle groups depending on patient’s symptoms. Muscle power is rated by the medical research council (MRC) system.

| Grade 0 | No flicker of contraction. | Grade 3 | Active movement against gravity without resistance |
| --- | --- | --- | --- |
| Grade 1 | Only a flicker of contraction. | Grade 4 | Active movement against gravity with resistance but less than normal |
| Grade 2 | Active movement with gravity eliminated. | Grade 5 | Full power |

***4. Reflexes***

Deep tendon reflexes- Basic technique

-Clearly identify tendon of muscle tested, position limb so it is at rest, strike tendon briskly and observe for muscle contraction and limb movement.

-Reflex trouble-shooting: Make sure patient is relaxed and that you are striking tendon rightly. Reinforcement (Jendrassik maneuver) helps to enhance reflexes. In leg, ask patient to pull hands apart while you strike. In upper extremities, ask patient to clench teeth.

*Biceps*: Place arm so it’s bent at 90 degrees. Place one of your fingers on the tendon and strike briskly.

*Triceps*: Arm can hang down at ninety degrees or on hips. Strike tendon directly.

*Brachioradialis*: Tendon for brachioradialis is approximately 10cm from wrist. Place arm so it is resting on patient’s thigh, bent at elbow. Strike firmly.

*Patellar*: Palpate patellar tendon and strike firmly.

*Achilles*: Hold foot at ninety degrees and strike tendon firmly.

Superficial reflexes

Plantar response: Gently stroke bottom of foot, starting laterally and near heel, moving up and across ball of feet (metatarsal head).

***5. Sensory testing***

Pain/Temperature and light touch

Pain: Use a sharp object (tooth pick). Ask patient to close eyes, start at top of foot. Orient patient by touching with sharp object, then non-sharp object (finger), defining sharp and dull. Touch lateral aspect of foot with both sharp or dull tool, and patient reports their response. Move medially across top of foot noting dermatomes.

Temperature: (not done routinely). Tested by running tuning fork under cold water and testing in same fashion like pain.

Light touch: This is also assessed across dermatomes using cotton wool. Upper extremities tested in similar fashion.

Proprioception

With one hand, grasp either side of great toe at interphalangeal joint. Place your other hand on the lateral and medial aspects of the great toe distal to the interphalangeal joint. Orient patient as to up and down. Flex the toe (pull it upward while telling patient what you are doing). Extend toe (pull it downwards while informing them which direction you are moving it). Ask patient to close eyes. Alternatively deflect toe up and down without telling patient in which direction you are moving it. Patient should be able to identify movement and direction. Do same for upper extremities.

Romberg test: Ask patient to stand still with feet as close together as necessary to maintain balance while eyes are open, and then eyes are closed. A loss of balance is an abnormal response. Be in position to catch patient in case he/she loses balance.

Vibratory Perception

Compress the ends of the 128MHz tuning fork just hard enough that the sides touch. Place the vibrating tuning fork on a bony prominence on the patient’s wrist or hand to be sure they recognize the vibration or ‘buzzing’. Compress ends of tuning fork again and immediately place gently but firmly on the top of the distal interphalangeal joint of one great toe and begin counting seconds. Instruct patient to tell you when vibration stops. Repeat three times and get the average vibration time. Do same for the other toe. If not felt, progress to proximal bony prominences (malleoli, tibial tuberosity).

***6. Coordination and gait***

Finger to nose testing (Testing for dysmetria): With patient seated, position your index finger at a point in space in front of patient. Instruct the patient to move their index finger between your finger and their nose. Reposition your finger after each touch.

Rapid alternating movements (Testing for dysdiadochokinesis): Ask patient to touch tips of each finger to the thumb of same hand. (Do for both hands). Direct patient to touch first the palm and then the dorsal side of one hand repeatedly against thigh.

Gait: -Ask patient to walk across room. Pay attention to balance, rate of walking and attitude of arms/legs. Ask patient to walk in straight line, putting the heel of one foot directly in front of the toe of the other (tandem gait).
